# Toward equity-oriented cancer care: a Strategy for Patient-Oriented Research (SPOR) protocol to promote equitable access to lung cancer screening

**DOI:** 10.1186/s40900-022-00344-y

**Published:** 2022-04-05

**Authors:** Ambreen Sayani, Jackie Manthorne, Erika Nicholson, Gary Bloch, Janet A. Parsons, Stephen W. Hwang, Bikila Amenu, Howard Freedman, Marlene Rathbone, Tara Jeji, Nadine Wathen, Annette J. Browne, Colleen Varcoe, Aisha Lofters

**Affiliations:** 1 Transition to Leadership Stream Postdoctoral Fellow at the Women’s College Research Institute, Women’s College Hospital, Toronto, Canada; 2 Canadian Cancer Survivor Network, Ottawa, Canada; 3 Canadian Partnership Against Cancer, Toronto, Canada; 4 St. Michael’s Hospital and Inner City Health Associates, University of Toronto, Toronto, Canada; 5 Wellesley Institute, Toronto, Canada; 6 Li Ka Shing Knowledge Institute, St. Michael’s Hospital, Toronto, Canada; 7 Department of Physical Therapy, Rehabilitation Sciences Institute, University of Toronto, Toronto, Canada; 8 MAP Centre for Urban Health Solutions, St. Michael’s Hospital, University of Toronto, Toronto, Canada; 9 Research Advisory Council, Lung Cancer Screening Strategy for Patient-Oriented Research Study, Women’s College Hospital, Toronto, Canada; 10 Arthur Labatt Family School of Nursing, Centre for Research on Health Equity and Social Inclusion, Western University, London, Canada; 11 University of British Columbia School of Nursing, Vancouver, BC Canada; 12 Department of Family and Community Medicine, Women’s College Hospital Family Practice Health Centre, University of Toronto, Toronto, Canada; 13 Peter Gilgan Centre for Women’s Cancers, Women’s College Hospital, Toronto, Canada

**Keywords:** Equity-informed patient-oriented research, Equity-oriented health care, Health equity, Health inequity, Lung cancer screening, Protocol, Patient engagement, Participatory co-design, SPOR

## Abstract

**Background:**

Screening for lung cancer with low dose CT can facilitate the detection of early-stage lung cancers that are amenable to treatment, reducing mortality related to lung cancer. Individuals are considered eligible for lung cancer screening if they meet specific high-risk criteria, such as age and smoking history. Population groups that are at highest risk of lung cancer, and therefore, the target of lung cancer screening interventions, are also the least likely to participate in lung cancer screening. This can lead to a widening of health inequities. Deliberate effort is needed to both reduce lung cancer risk (through upstream interventions that promote smoking cessation) as well as midstream interventions that promote equitable access to lung cancer screening.

**Methods:**

This protocol paper describes an equity-informed patient-oriented research study. Our study aims to promote equitable access to lung cancer screening by partnering with patients to co-design an e-learning module for healthcare providers. The learning module will describe the social context of lung cancer risk and promote access to lung cancer screening by increasing equity at the point of care. We have applied the Generative Co-Design Framework for Healthcare Innovation and detail our study processes in three phases and six steps: Pre-design (establishing a study governance structure); Co-design (identifying research priorities, gathering and interpreting data, co-developing module content); and Post-design (pilot testing the module and developing an implementation plan).

**Discussion:**

Patient engagement in research can promote the design and delivery of healthcare services that are accessible and acceptable to patients. This is particularly important for lung cancer screening as those at highest risk of developing lung cancer are also those who are least likely to participate in lung cancer screening. By detailing the steps of our participatory co-design journey, we are making visible the processes of our work so that they can be linked to future outcomes and related impact, and inform a wide range of patient co-led processes.

## Plain English summary

Lung cancer is the most commonly diagnosed cancer in Canada and is responsible for a quarter of all cancer-related deaths in the country. Screening for lung cancer using tools such as a CT scan can allow us to find lung cancers when they are still small and curable. People can receive a lung CT scan depending on how old they are and for how long they have smoked cigarettes. Certain groups of people, particularly those who have fewer resources such as time and money, and those who experience injustice because of who they are and how they look are less likely to participate in lung cancer screening. We can increase participation in lung cancer screening by educating healthcare providers on appropriate and timely ways to talk to patients about lung cancer screening. In this paper, we outline the steps of a patient-partnered study in which many different stakeholders such as patients, providers and policy-makers have come together with a goal to improve equity in access to lung cancer screening. We will do this by jointly creating an online learning module that will educate healthcare providers on how life experiences shape smoking behaviour and lung cancer risk. The module will also impart key skills on how to deliver care which is timely, appropriate and safe. Once the module is ready it will be freely available to all healthcare providers to support the fair and just delivery of lung cancer screening in the province of Ontario and elsewhere.

## Background and rationale

Lung cancer is the most commonly diagnosed cancer in Canada and it is estimated that one in fifteen Canadians will be diagnosed with lung cancer over their lifetime [1, 2]. Lung cancers are usually detected at an advanced stage (stage III or stage IV) when the chances for curative therapy are quite low. In Canada, lung cancer contributes to a quarter of all cancer-related deaths [1].

The incidence and mortality rates of lung cancer closely follow patterns in the smoking epidemic, such that both a rise and fall of the rates of smoking consumption precedes the subsequent rise and fall of lung cancer incidence and mortality by about 20 years [1, 3]. It is for this reason that smoking cessation programs, as well as innovative methods to detect early stage lung cancer, have been identified as a national priority in the Canadian Strategy for Cancer Control, 2019–2029 [4]. As of April 2021, lung cancer screening (LCS) is being offered through an organized public health program in Ontario called the Ontario Lung Screening Program. Screening is currently offered at four sites across the province: The Ottawa Hospital, Health Sciences North in Sudbury, Lakeridge Health in Oshawa and the University Health Network in Toronto [5]. The objective of the program is to detect lung cancers that are asymptomatic and potentially curable, thereby reducing lung cancer related mortality [6].

As with any cancer screening, it is important to take into consideration the population distribution of disease so that people at greatest risk of developing lung cancer are the ones specifically targeted for the LCS intervention [7]. Accordingly, individuals between the ages of 55–74 years, who have smoked daily for a period of 20 years, are currently eligible for referral [5]. An inherent assumption in the current eligibility criteria is that age-eligible individuals who have smoked or are currently smoking will be able to access the screening program and be ready to participate in lung cancer screening. Contrary to this, evidence points to the unequal burden of lung cancer risk which is shaped by the social patterning of smoking behaviour and smoking cessation which is skewed so that individuals with lower levels of education, less income and in a lower occupational class are more likely to be smoking [8–10] and less likely to be successful at smoking cessation [11, 12] Further to this, there are well documented and significant inequities in access to cancer screening in populations based on differences in gender, race, ableism, social class and rural location [13–15]. In Canada, structural barriers such as systemic racism and other forms of discrimination, historical injustice and stigma contribute to the inequitable participation in lung cancer screening for First Nations, Inuit and Métis, recent immigrants and those living in conditions of poverty and precarious housing [16, 17].

To prevent a widening of health inequities as a result of the unequal uptake of lung cancer screening interventions, it is important to reallocate resources to meet the needs and priorities of populations experiencing the most inequities [18]. This approach, called a ‘priority population’ approach [18–20] is ‘regardful’ of structural inequalities which shape disease-risk and access to care [21, 22] and is ‘responsive’ to the needs of patients [23]. Clinical encounters which are equity-oriented and trauma- and violence-informed can positively influence peoples’ decision to participate in LCS [17]. On the other hand, the provision of care that is stigmatizing, lacks self-reflexivity and perpetuates personal and systemic biases can create unsafe spaces that discourage participation in LCS [17]. In Canada, this is of heightened importance as family physicians are gatekeepers to LCS [5] and other primary and community care providers such as nurse practioners, nurses, social workers, dieticians, community and peer support workers, health promoters, and occupational therapists are strategically placed to provide the wrap around support services that can enable timely referrals by physicians [24].

Our work to date has demonstrated a disconnect between the needs and priorities of individuals who are at a high-risk of developing lung cancer [17] and the perceptions of need and clinical care imparted by primary care providers, particularly for individuals living in marginalizing social conditions [24]. Programs that support decision-making for cancer care including screening, and policies that support the timely adoption of findings that seek to reduce inequities in care for priority populations are strategic priorities for the American Association of Cancer Research [18] and the Canadian Institutes of Health Research (CIHR) Institute for Cancer Research [19].

Accordingly, we propose to bridge this research-to-practice valley [25] by partnering with relevant stakeholders, including policy-makers, healthcare providers and patients (defined as individuals with relevant lived/living experience) in the co-design [26] of healthcare innovations that are scalable, effective and reflect the needs and priorities of all stakeholders [27]. Central to the process of participatory co-design is engagement with members of community who have relevant lived/living experience so that they are involved in the co-production of processes and project decision-making [28]. Given the inequitable distribution of lung cancer risk and access to care across the population it is important to proactively engage with communities that are structurally underserved and seldom-heard because of exclusionary institutional systems and discriminatory research designs [29]. This equity-oriented approach (EOA) to patient engagement [29] can integrate the worldview of patients into healthcare innovation co-design and promote the adoption of equitable healthcare practices [25, 27].

This article describes the protocol for a CIHR-funded Strategy for Patient-Oriented Research (SPOR) study. We are adapting the Generative Co-Design Framework for Healthcare Innovation [27] to our study context and describe our work in three stages: Pre-design (September 2019 to May 2021); Co-design (May 2021 and currently ongoing) and Post-design (expected study outcomes by May 2024). By sharing our study protocol for a patient-oriented research (POR) healthcare innovation co-design, we are responding to recent calls for documentation of participatory co-design so that linkages between process, outcomes and impact can be better understood [26, 28].

### Objective

The objective of this CIHR-funded SPOR study is to enhance the delivery of equitable primary care and consequent access to lung cancer screening for priority populations by partnering with patients to co-design a learning module for healthcare providers. The learning module will illuminate the social context of lung cancer risk and promote the delivery of self-reflexive equity-oriented care.

### Approach

Learning tools that support initial and continuing professional development are a key form of practice-focused knowledge mobilization [30]. Interventions that include a mix of e-learning components and organizational strategies to enhance equity at the point of care, for example, have shown to improve care providers’ confidence and abilities to provide Equity-Oriented Health Care (EOHC) in primary health care settings [31]. An approach to EOHC used in the health equity research program known as “EQUIP” (see Box 1) includes three key dimensions described below: trauma- and violence-informed approaches to care (TVIC), cultural safety, and harm reduction through substance use health [32]—emphasizing knowledge about trauma, understanding of the context of people’s lives, and explicit attempts to build trust—both at the point of care and at the level of organizational practices [31].

**Box 1:** Key definitions

Equity-Oriented Health Care (EOHC): The EQUIP Healthcare Model
An approach to care that considers the effects of structural inequities, including the inequitable distribution of the determinants of health (such as poverty, lack of affordable housing); the impact of intersecting forms of racism, discrimination and stigma (e.g., related to mental illness, substance use, non-conforming gender identities, etc.) on people’s access to services and their experiences of care; and the frequent disconnect between usual approaches to care and the needs of people who are most affected by health and social inequities (Cited with permission from Browne et al., 2018, p. 2)
EQUIP’s “take” on Equity-Oriented Health Care incorporates the three key dimensions, listed below, which overlap and can be tailored to any health care setting
Trauma- and violence-informed care (TVIC):
Recognizing and limiting the effects of trauma and violence, including structural violence, on peoples’ lives, health and care experiences
Cultural safety (CS):
The practice of actively reducing power imbalances, systemic racism, and discrimination in clinical encounters
Harm reduction:
A focus on preventing harms from substance use and intersecting forms of stigma, and attention to the notion of substance use health, as the achievement of self-defined goals of well-being across the continuum of substance use ranging from no substance use to substance use disorder. Providing substance use health care requires a) deprioritizing abstinence as the primary success outcome of health care, b) removing barriers to care, including intersecting forms of stigma, and c) facilitating access to social determinants of health for those with limited access [29]

In research conducted by the EQUIP team in primary health care settings, providing more EOHC was predictive of improved self-reported health outcomes across time for people living in conditions of social marginalization [33]. This was achieved by enhancing patients’ comfort and confidence in their care and their own confidence in preventing and managing health problems [33]. Interventions to improve the delivery of EOHC based on this understanding are currently undergoing evaluation in emergency department settings [34, 35]. As a person-led and -centred approach that encourages providers to actively listen and build on a person’s strengths, needs and agency to determine best next steps in care [36], these approaches have shown promise as a way to shift individual practice, and organizational policies, towards safer and more equitable care [37, 38].

In the context of LCS, increasing health providers’ knowledge about trauma (such as the impact of trauma on social patterning of smoking addiction), the health effects associated with experiences of trauma and violence (including experiences of stigma and multiple forms of discrimination, including racism, ableism and classism, that shape access to care) and developing skills to apply the principles of TVIC, cultural safety and harm reduction in practice can promote the delivery of equity-oriented, person-centred and safe clinical encounters leading to a higher uptake of preventative healthcare practices such as lung cancer screening.

## Methods

**Fig. 1:**
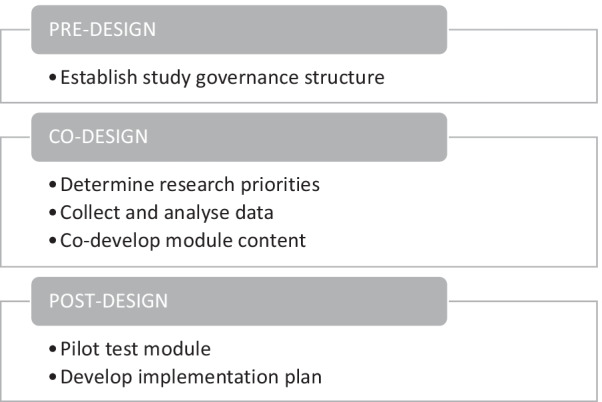
Generative Co-Design Framework for Healthcare Innovation applied to co-develop a healthcare provider facing e-learning module to promote equitable access to lung cancer screening

We have applied the Generative Co-Design Framework for Healthcare Innovation to guide our study processes in three phases and six steps: Pre-design (establish a study governance structure); Co-design (identify research priorities, gather and interpret data, co-develop module content); and Post-design (pilot test the module and develop an implementation plan) (Fig. 1).

### Pre-design

Pre-design refers to the preparation for participatory co-design. In this study we are following the SPOR Patient Engagement Framework [39] and applying principles of equitable patient engagement by prioritizing the co-building of safe spaces, addressing issues of accessibility and building capacity through trusted relationships [29]. We have partnered with patients to establish governance structures and have received ethics approval from Women’s College Hospital Research Ethics Board.

#### Step 1: Establish study governance structure

The study is governed by a Research Stakeholder Council (RSC) (a collaborative group of stakeholders who oversee study rigour and drive applicability of the study based on provincial and national level priorities) and a Research Advisory Council (RAC) (a patient partner working group who is steering the direction of the study). As a research team, the RSC and RAC are applying principles of integrated Knowledge Translation (iKT) [40] to co-develop research questions, guide study methodology, collect data, interpret findings, disseminate results and have co-authored this paper.

The RSC includes stakeholders involved in the design, delivery, accessibility and uptake of LCS at the Canadian provincial (AL) and federal levels (EN); healthcare providers with expertise in care for priority populations (SH) and the social determinants of health (GB); health service researchers with methodological expertise in patient-oriented research (JP) and equitable patient partnerships (AS). The RSC patient partner (JM) leads a pan-Canadian network of cancer patients, families, survivors, friends and community partners. The RSC was established prior to applying for grant-funding in September 2019. Since receiving grant funding (May 2020), the RSC meets every three months to guide the research study for relevance based on patient-need, applicability based on emerging guidelines and health system-level priorities.

When the RSC first convened, a key priority was to facilitate the creation of a group of 3–4 patient partners who could bring expert knowledge based on their diversity of lived experiences to the co-design process. In March 2020, the COVID-19 pandemic was declared and healthcare systems were operating in emergency mode, bringing almost all patient engagement efforts in Canada to a pause [29]. Already embedded in a culture of tokenistic patient engagement practices and exclusionary institutional structures [29], the RSC needed to pivot and innovate to engage seldom-heard members of community. Respectful partnership with communities who have been historically and structurally disempowered requires an equity-oriented, culturally-safe and trauma- and violence-informed approach to engagement, which is sustainable beyond the life-cycle of any single research study [29]. This led to a period of deep listening and learning from structurally underserved members of community and the co-design of a sustainable patient-partnered model of diverse patient engagement called Equity-Mobilizing Partnerships in Community (EMPaCT), (June 2020–March 2021). EMPaCT is an independent community table made up primarily of patients/diverse members of community who conduct health equity assessments based on their intersectional lived experiences of social and structural inequality. Researchers and other decision-makers consult with EMPaCT to learn how to make their projects more inclusive and equitable, details of which can be read elsewhere [41].

Participatory co-design that attends to power dynamics and partners with patients in ways that are meaningful requires a commitment to learn from members of community and the building of responsive practices. Relationship building and establishing trust, thus, forms the foundation of participatory co-design and resources (time, money, human capital) are needed to adequately support this. EMPaCT enabled us to nurture already existing relationships and build new relationships of trust with members of community paving the way to establish the study RAC (April 2021).

The RAC is a self-governing council formed by four patient partners residing in Ontario who offer expertise developed from diverse experiences intersecting across elements of race, gender, disability, Indigeneity, immigration, poverty and homelessness. Members of the RAC (BA, HF, TJ, MR) meet with the study Principal Investigator (PI) (AS) once a month as agreed in consensus. This structure creates a space where the members of the RAC and the PI can nurture relationships of trust and engage in authentic dialogue for co-learning [42]. The meetings are held virtually due to the COVID-19 pandemic. To support equitable participation, meetings are held at a time and date accessible to everyone. RAC members receive compensation for hours worked on the project according to standards set by SPOR [39] and digital devices to facilitate virtual participation as needed. Creating non-hierarchical safe spaces where all patient partners share a sense of purpose, directing the design and aims of the research study, and building capacity through partnerships are RAC guiding principles [29, 39].

**Fig. 2:**
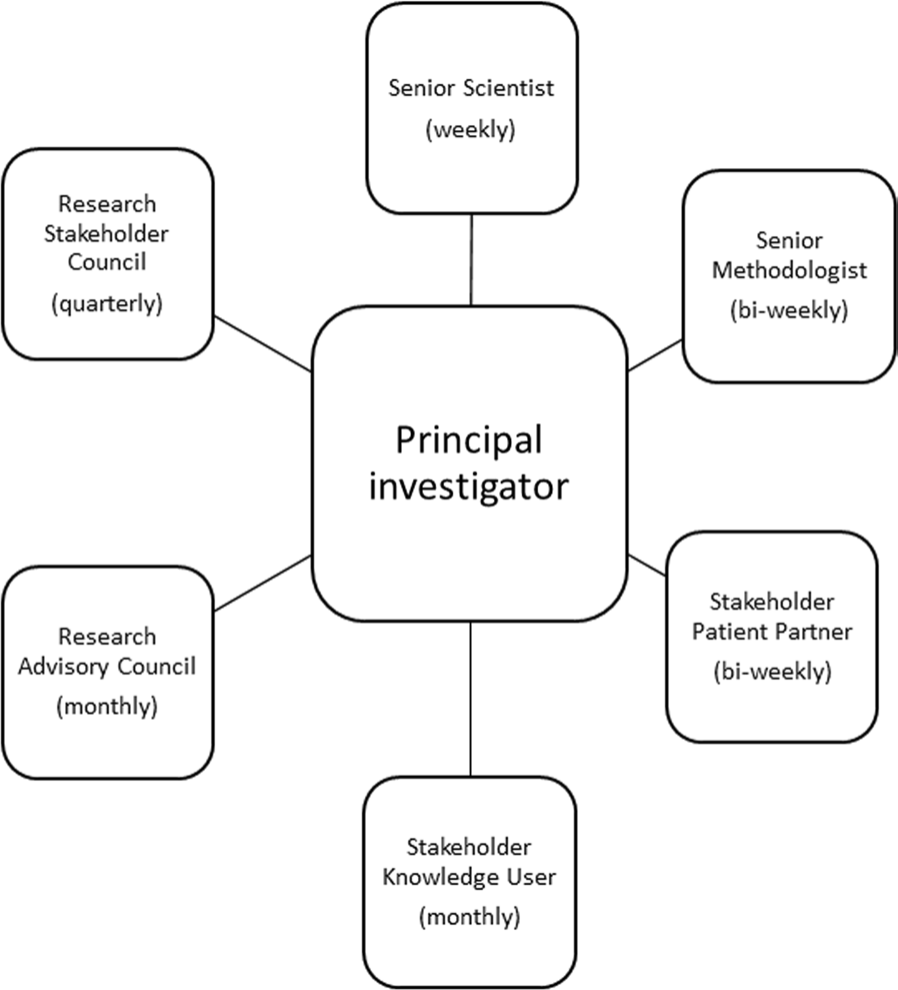
Study governance structure: at the core of the governance model is the study PI who builds consensus with all patient partners and study stakeholders through engagement in multiple safe spaces

Figure 2 shows our study governance structure. At the core of the governance model is the study PI who has regular touchpoints with all patient partners and study stakeholders. This structure has facilitated the creation of multiple spaces of co-learning through which the study PI weaves together knowledge, builds consensus for next steps and reports back regularly to the study RSC (which includes the stakeholder patient partner, senior methodologist, senior scientists and knowledge user). This structure has enabled the study team to respond in an agile and iterative way to research priorities as they emerge.

### Co-design

The involvement of patients in all phases of the research spectrum such as setting study priorities, identifying methods of data collection, interpreting findings and disseminating results, can increase the ‘real-world’ relevance and applicability of research evidence [43]. While the focus of most patient-oriented work to date has been to enhance applicability of findings for patient-facing materials, services and guidelines [44], our approach is novel in that we are partnering with patients to co-design *health professional-facing* learning materials. Patients are defining how they want to receive care and developing tools to educate healthcare providers on what matters to them, when it matters and how they would like to be approached for potentially stigmatizing conversations.

#### Step 2: Identify research priorities

To date, RAC members have collaborated with the study PI to identify research priorities (April 2021–August 2021), held a meeting with the RSC (September 2021), and strategized ways to disseminate the learning module once it is ready (October 2021) (see Table 1). In subsequent meetings RAC members will be involved in co-designing the research tools (including interview guides), participant recruitment strategies and analyzing interview data. RAC members will co-design the learning module content together with the RSC.

**Table 1:** Steps involved in research priority setting and participatory co-design timelines

Topic	Initial idea	Discussion point	Consensus
Research focus (April–May 2021)	To understand the equity-oriented learning needs of family physicians in Ontario to inform the co-design of a learning module	Is there is a need to focus on all family physicians in Ontario, or would it be better to speak to family physicians who are more likely to be providing care to priority populations?	To understand the equity-oriented learning needs of family physicians in community-focused practice settings in Ontario in order to inform the co-design of a learning module
Research question (June 2021)	To understand learning needs of family physicians as they are gatekeepers to lung cancer screening	Is there a need to speak to just family physicians? The wrap around support services offered by all primary care providers in community-focused practice settings create the environment and support structures needed for participation in LCS	Research question to include all frontline primary care providers in community-focused practice settings: To understand the learning needs of primary care providers in community-focused practice settings in Ontario in order to inform the co-design of a learning module
Research approach (July–August 2021)	To interview healthcare providers in community-focused practice settings to inform the co-design of an equity-focused learning module to support lung cancer screening uptake	Can we gather more informative data from providers in community-focused practices settings who have previously received equity-focused training as they have the relevant prior learning and clinical experience to be able to contextualize LCS?	To conduct semi-structured interviews with primary care providers in community-focused practice settings who have received prior training on how to deliver equity-oriented healthcare (EOHC) in order to understand the equity-oriented skills they apply in practice to promote equitable access to LCS
Dissemination strategy (October 2021)	To co-design a learning module that will promote the delivery of EOHC to increase uptake of LCS in priority populations	Can we co-design a module to be included with other EOHC learning material rather than building a totally new standalone module?	To partner with Equipping Health & Social Services for Health Equity (EQUIP) to co-develop and disseminate the learning module on the EQUIP web platform so that it can be widely and freely accessible to learners in Ontario and elsewhere

#### Step 3: Gather and interpret data

The perspectives of primary care providers working in community-focused practice settings on access to LCS for priority populations will be used to inform the thematic content of the learning module. To do this, we will recruit and interview primary care providers (PCPs) through primary care practices in Ontarioin Ontario. We define PCPs as physicians, nurse practitioners, nurses, social workers, dieticians, community and peer support workers, health promoters, occupational therapists and other relevant professions who work in community-focused practice settings. We will recruit PCPs that have received prior training or self-directed learning in equity-oriented or trauma- and violence-informed care either through workshops, or online modules such as those offered by EQUIP Healthcare. These PCPs have the most relevant prior training to inform the development of a new module focused on LCS and they are most likely to be in clinical encounters with patients who are eligible. From our previous experience in similar projects, it is anticipated that approximately 10–15 PCPs will be interested in participating.

Semi-structured interviews will be conducted using an interview guide co-designed with members of RAC. The interview guide will contain questions about lung cancer risk, access to preventative care such as LCS, and ways to navigate potentially stigmatizing clinical encounters. Speaking to PCPs who have received prior equity-oriented training will enable us to understand how they are applying those skills in practice and what adaptations are needed to support equitable access to LCS. Interviews will be conducted by the PI and data will be anonymized before sharing with the research team (including the members of RAC). We will use an iterative thematic analysis approach to identify common themes and patterns of meaning across the data set [45]. A flexible coding structure will be developed to allow for the creation of additional or “free” nodes when new emerging ideas or themes are identified. Theoretical saturation, trustworthiness and validity checks will provide assurance of data quality and rigor [46]. Data management will be facilitated using NVivo software (version 12).

#### Step 4: Co-design module content

Using a process of co-generative inquiry [47] we will weave together thematic content from the interviews with the lived experiences of the members of RAC and co-create the content material for the learning module. Such materials will include, videos, case studies, a learner’s notebook, and end-of-module assessments. We will co-produce video narratives with each stakeholder group of the study as shown in Fig. 1 (i.e., patient partners, including members of RAC, knowledge users, healthcare providers and physicians) and record videos showing the perspectives of each stakeholder. The case studies will be based on the lived experiences of patient partners including scenarios where EOHC/TVIC can lead to safe clinical encounters and person-centred, equity-oriented care. Additional materials will include a learner’s notebook (a downloadable workspace containing key concepts, additional activities and prompts to enter personal reflections), and end-of-module assessments to test learners’ knowledge, attitudes and skills.

### Post-design

For the purposes of our work, we follow on the definition of post-design by Bird et al. [27]. Accordingly, the post-design phase will consist of checking the outcomes of our participatory co-design (the learning module) with all stakeholders and making adaptations to ensure relevance and appropriateness of the final outcome. We will also co-identify plans for knowledge mobilization and implementation based on stakeholder dissemination priorities.

#### Step 5: Pilot test module

The co-designed learning module content will be hosted and freely available on the EQUIP website at the University of British Columbia. To do this, we will develop a curriculum platform on HTML 5 Package (H5P is a freely available software which allows educators to create interactive and engaging video content) and mount the curriculum on Canvas Learning Management System (LMS software is a digital learning management system that allows educators to create and present online learning materials and assess student learning). Standard EQUIP designed evaluation and feedback questions will be tailored to the LCS-focused module.

We will invite study stakeholders (including patient through the members of RAC) to check the relevance and appropriateness of the online module once it is ready. Select primary care providers and health system stakeholders will also be invited to provide feedback on the LCS module through an online questionnaire. We will make modifications to module design as needed based on stakeholder and user-identified preference.

#### Step 6: Develop implementation plan

We will jointly identify implementation goals and determine implementation evaluation criteria including Patient-Reported Outcomes Measures (PROMs) for effective LCS which reflect the needs and priorities of all stakeholders. It is likely that the different stakeholders will have different definitions of successful implementation outcome measures, such as cost-effectiveness, ease of access, and utilization measures of LCS. It will be our objective to create a congruent implementation plan [48] by identifying outcome measures that are salient to all stakeholders in order to promote applicability and validity of the learning module across a range of primary care practices in the province of Ontario.

## Discussion and anticipated impact

Patient engagement in research can lead to better health outcomes by reprioritizing healthcare decision-making to match the needs and concerns of patients [39]. In particular, access to care and acceptability of care provided can be increased by partnering with patients to inform how services and care are delivered. The key to designing healthcare services that work for everyone is to partner with those who are least likely to be included in decision-making and most likely to experience a higher burden of illness and inequitable health outcomes [29]. This can be done by using equity-oriented approaches to developing and nurturing relationships and by building capacity through safe, trauma- and violence-informed resources and processes [29].

Our work to date, and our planned work, are deliberate efforts to shed light on some of the processes involved in conducting Equity-Informed Patient-Oriented Research (EI-POR). The actual steps involved in building equitable patient partnerships must be rooted in relationships of trust that take time, commitment, an array of partnership and communication skills, and adequate funding that recognizes the often invisible effort and value of this work [29]. We have detailed our pre-design process, including how various stakeholders at different levels of decision-making (patients, providers, policy-makers, funders) have come together with a unified goal: to promote equitable access to lung cancer screening. We have described our pathway to forming the study governance structure, including how the members of the RAC came together, how frequently they meet and how capacity has been built to enable equitable partnerships. We have also shared a step-by-step evolution of how our research priorities emerged and recounted how we have moved forward with the study goals in a reflexive and participatory way.

As next steps in our work, we are co-designing our research tools and will collect data. Co-generative analysis of our research findings will inform the content of the learning module, which in turn will be ready for dissemination in partnership with EQUIP. Our implementation plan will tie together the goals and salient outcome measures from across all our study stakeholders and we will be ready for implementation across primary care settings in Ontario to support equitable access to LCS. Once our module has been implemented and evaluated in Ontario, it can be spread and scaled at a national and international level.

We are aware that learning modules in isolation will do little to influence the structural barriers to care experienced by patients as a result of social inequities, nor will learning modules alone challenge underlying economic systems that pattern smoking behaviour and lung cancer risk. Learning modules, however, can promote the delivery of equity-oriented care at the point of care resulting in a safer clinical encounter [33, 49], support PCPs to contribute to structural changes in how LCS is organized and delivered, and promote advocacy for equity-oriented policies across sectors. These, is turn, can influence the choice to participate in LCS [17]. Our work on equity-oriented cancer care will seed the beginnings of a collaborative and interdisciplinary network of stakeholders who are designing organizational processes to support equitable access to cancer care and advocating for systems-level policy change.

## Data Availability

Not applicable.

## Abbreviations

CIHR: Canadian Institutes of Health Research
CHC: Community Health Centre
CT scan: Computed tomography scan
EI-POR: Equity-informed patient-oriented research
EQUIP: Equipping Health & Social Services for Health Equity
EMPaCT: Equity-Mobilizing Partnerships in Community
EOA: Equity-oriented approach
EOHC: Equity-Oriented Health Care
iKT: Integrated Knowledge Translation
LCS: Lung cancer screening
PCP: Primary care provider
PI: Principal investigator
PROM: Patient-Reported Outcomes Measure
RAC: Research Advisory Council
RSC: Research Stakeholder Council
SPOR: Strategy for Patient-Oriented Research
TVIC: Trauma- and violence-informed approaches to care
